# Oxygen Consumption of the River Nerite *Theodoxus fluviatilis* in Different Salinities

**DOI:** 10.1002/jez.70033

**Published:** 2025-10-07

**Authors:** Laura I. R. Fuchs, Johanna Klepsch, Pia Katzberg, Christian Müller, Jan‐Peter Hildebrandt

**Affiliations:** ^1^ Animal Physiology and Biochemistry, Zoological Institute and Museum University of Greifswald Greifswald Germany

**Keywords:** changes in environmental salinity, ecotypes, neritid snail, osmotolerance, respirometry

## Abstract

The river nerite *Theodoxus fluviatilis* (Linneaus, 1758) is an euryhaline osmoconformer found in freshwater (FW) and brackish water (BW) habitats across Europe and western Asia. In northern Germany, *T. fluviatilis* forms regional subgroups, the FW and the BW ecotypes. Members of these ecotypes differ in shell morphology and in shell size as well as in their metabolic pathways of accumulating organic osmolytes under hyperosmotic stress. Oxygen consumption rates were measured, as a noninvasive fitness‐indicator, of animals exposed to external media with different salinities. The BW water snails were exposed to hyper‐ and hyposaline conditions, while the FW snails were naturally only exposed to hyperosmotic conditions. Respiration rates in specimens of the BW ecotype stayed more or less constant overall in all salinities tested. While the BW snails' respiration rates were not affected by the salinity treatment per se, they were affected by the sequence of salinity treatments that they were exposed to (hyperosmotic conditions first vs. hypoosmotic conditions first). Respiration rates strongly declined in the FW ecotype individuals with increasing medium salinity. We suggest that the hypersaline conditions pose greater stress for the animals than the hyposaline conditions because of the higher metabolic activities required for tissue volume regulation.

## Summary

- The river nerite *Theodoxus fluviatilis* (Linneaus, 1758) is an euryhaline osmoconformer.
- Oxygen consumption rates of animals exposed to external media of different salinities were measured.
- Differences between ecotypes became apparent.

## 1 Introduction

Species inhabiting coastal environments close to the water surface or in the tidal zone are exposed to fluctuations in environmental factors, such as temperature, UV radiation, and salinity (Velasco et al. 2019). Salinity fluctuations are expected to become more frequent and extreme as climate change progresses. This includes the salinization of coastal water bodies, while increased precipitation may lead to the freshening of shallow marine and brackish water (BW) habitats (Reid et al. 2009). Salinity has been shown to have profound effects on the physiology of aquatic invertebrates, as well as limiting effects on the survival and distribution of osmoconforming species, as the energy metabolism of such species responds to changes in internal osmotic or ionic concentrations (Ou et al. 2021; Pascual and Drake 2008; Qin et al. 2020). Balancing of the internal and the external osmotic concentration is required under changing external salinity conditions to avoid cell‐ and tissue shrinkage or ‐swelling, and the underlying mechanisms need additional energy (Shumway 1977).

The river nerite *Theodoxus fluviatilis* (Linneaus, 1758) is an euryhaline osmoconformer found in freshwater (FW) and BW habitats across Europe and western Asia (Anistratenko 2005; Bandel 2001; Bunje and Lindberg 2006; Zettler et al. 2004). It lives in shallow waters on hard substrate. *Theodoxus* uses its radula to crack the cell envelopes of diatoms, their main food, using the hard substrate as an abutment (Zettler et al. 2004). The spatial distribution of *T. fluviatilis* is limited by its reproductive mode and the slow pace at which these gastropods move, making it vulnerable to variations in salinity. Juvenile snails hatch directly from an egg sack deposited by the female. Since there is no free‐swimming larval stage, the distribution pace and the speed of range expansion are rather limited (Bondesen 1940; Kirkegaard 2006).

According to COI gene analyses, *T. fluviatilis* is thought to have originated in BW habitats and secondarily invaded FW habitats (Bunje 2005; Bunje and Lindberg 2006). In northern Germany, *T. fluviatilis* forms regional subgroups, the FW and the BW ecotypes, respectively. Members of these ecotypes differ in shell morphology and in shell size (Glöer and Pešić 2015; Kangas and Skoog 1978; Wiesenthal et al. 2018; Zettler et al. 2004) as well as in their metabolic pathways of accumulating organic osmolytes under hyperosmotic stress (Wiesenthal et al. 2019). FW individuals accumulate free amino acids by hydrolyzing storage proteins, while BW animals seem to newly synthesize amino acids. In addition to amino acids, BW animals under hyperosmotic stress accumulate substantial amounts of urea, while the FW animals do so only moderately. Only the BW animals are able to regulate the expression of DUR3‐like urea transporters in response to changes in the external salinities (Knobloch et al. 2023). However, there is as yet no indication that *T. fluviatilis* is able to regulate abundances or activities of ATPases (Na^+^/K^+^‐ATPase or V‐ATPase) in response to salinity changes (Knobloch et al. 2022).

Although *Theodoxus* does not seem to invest substantial amounts of energy in osmoregulation, changes in ambient salinity may affect the energy metabolism of the animals due to the need for volume regulation (degradation/excretion or accumulation of organic osmolytes) and to keep up a certain degree of motility that is required to obtain food. Typically, the respiration rate in euryhaline osmoconforming species stays stable over a wide range of salinities (Svetlichny et al. 2012), but tends to decrease towards the upper and lower salinity limits of the given species (Sander and Moore 1978). To investigate whether *T. fluviatilis* exhibits similar behavior, a noninvasive fitness indicator was assessed by measuring the oxygen consumption of individuals exposed to external environments with varying salinities. Previous studies (Wiesenthal et al. 2018) revealed that being exposed to high salinities is stressful to members of the FW ecotype. Thus, we expected that these animals lower the rate of oxidative metabolism under such conditions. The BW ecotype individuals were expected to keep consistent respiration rates over the entire range of salinities tested, with decreasing rates only in the salinity ranges close to the upper (28 PSU) or lower (0.5 PSU, Wiesenthal et al. 2018) limits. Understanding species‐specific tolerance thresholds and adaptive mechanisms, such as phenotypic plasticity, is crucial for predicting biodiversity shifts and implementing conservation strategies in vulnerable environments such as coastal and estuary habitats.

## 2 Materials and Methods

### 2.1 Experimental Animals

Adult snails were collected at three FW sites (S1−S3) and one BW (S4) location in north‐eastern Germany (Wiesenthal et al. 2018). Water temperature and salinity were recorded at each sampling site. The experimental animals were kept in small glass aquariums (6−10 L) with constant aeration, furnished with substrate, rocks, and water from their original habitats. The rocks were covered in algae and diatoms serving as a food source for the snails. Additionally, the diatoms *Nitschzia* sp. and *Planothidium* sp. were cultivated, and aliquots of the suspensions were transferred to the aquariums on a regular basis. The aquariums were kept in climate chambers with a 12 h:12 h light:dark cycle and the temperature was slowly adjusted to 18°C, by increasing the climate chamber temperature by 1°C per week. The snails were kept in the laboratory for no longer than 6 months before the experiments. Medium osmolality was frequently controlled using a Vapro 5520 osmometer (Wescor Inc., Logan, Utah, USA).

### 2.2 Diatom Cultures

The benthic diatoms *Nitzschia* sp. and *Planothidium* sp. were cultivated in aerated 500 mL Erlenmeyer beakers under a 12 h:12 h light:dark cycle at 15°C in Guillards f/2 marine water enrichment solution (Sigma Aldrich, Merck KGaA, Darmstadt, Germany) diluted with artificial sea water (prepared by dissolving “Tropic Marine Classic” sea salt [Dr. Biener GmbH, Wartenberg, Germany] in deionised water) at salinities of 15 PSU and 0.5 PSU. Before the start of the respirometry experiments, glass beakers, containing pebbles and the corresponding experimental medium salinity, were inoculated with both *Nitzschia* sp. and *Planothidium* sp. The pebbles were subsequently covered by diatom growth and later used in between experimental setups to supply the snails with food ad libitum.

### 2.3 Respirometry—Experimental Set Up

The experimental setup was placed in a climate chamber with a constant temperature of 18°C and a 12 h:12 h light:dark cycle. Respirometric measurements were conducted using the Q‐Box AQUA Aquatic Respirometry Package (Qubit Systems Inc., Kingston, ON, Canada). The intermittent‐flow system was operated with two experimental chambers (1.25 mL volume) running in parallel. The experimental chambers were placed in a plastic aquarium containing 8 L of constantly aerated medium. The medium was prepared, a day before the start of an experiment, by adding “Tropic Marine Classic” sea salt (Dr. Biener GmbH, Wartenberg, Germany) to deionised water. Intermittent‐stop‐flow respirometry is divided into two phases: the measurement phase and the flush phase. During the measurement phase, the chamber is closed and the depletion of oxygen is measured by optical dissolved oxygen probes (DO probes). The duration of the measurement phase was adjusted (30−120 min) depending on the size/weight and metabolic activity of the experimental animals. The aim was not to allow more than a 50% decrease in oxygen concentration during one measuring cycle, since the oxygen depletion of the medium can influence the respiration rate of the animals (Berg and Ockelmann 1959; Cheung et al. 2008; Lumbye 1958). The measurement phases were interrupted by flush phases, during which the medium in the chambers was replaced by fresh, aerated medium. The flush phase duration was set to 3 min each.

Experimental animals were sexed a day before the experiment and kept in beakers containing the respective starting medium (12 PSU or 0.5 PSU) and diatom‐overgrown pebbles. Shortly before the start of the experiment, the snails were weighed, and the shells were cleaned of most of the biofilm using cosmetic tissues to minimize background respiration. One snail was placed into each of the submerged respirometry chambers situated in the tank with surrounding aerated medium. Movement of the snails within the chambers could not be controlled. However, the experimental chambers were quite small, with a volume of 1.25 mL, each, which did not allow the animals to move around excessively. The respiration measurements were run for 24 or 48 h. Initially, the duration was set to 48 h, in case the snails would take time to open the operculum and start moving, after the transfer to the new salinities. However, we discovered that snails would typically start moving quite soon after the transfer; therefore, the duration was decreased to 24 h. Upon completion of each measurement period, the snails were taken out of their chambers and placed back into beakers filled with medium at the salinity corresponding to the experimental salinity and diatom‐overgrown rocks. The total experimental duration for all salinities tested ranged from 8 to 16 days; therefore, the snails were fed ad libitum in between salinity treatments to ensure that the snails remained in a nutritional steady state with prandial oxygen consumption being constant over the experimental period (Von Brand et al. 1948).

Background respiration was measured in the empty chambers after each experiment for three or more consecutive stop‐flow cycles to account for oxygen consumption not caused by the animal itself.

**Figure 1:**
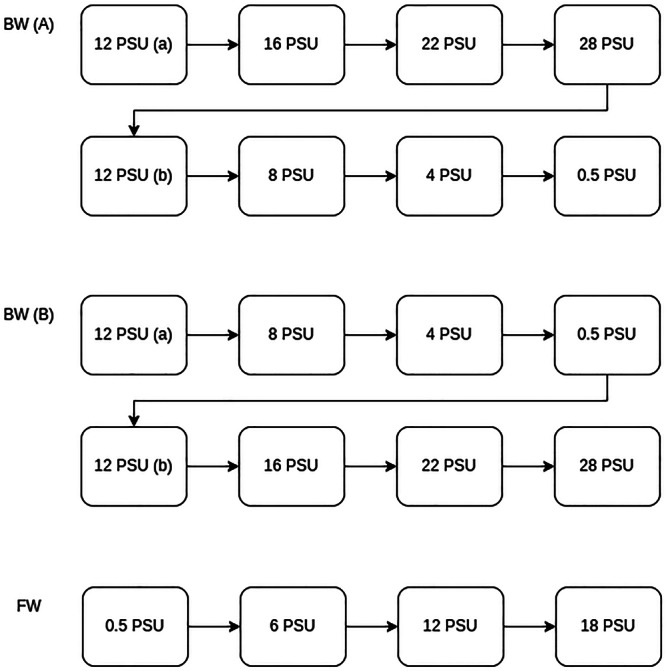
Experimental design for the three different treatment groups: BW (A) is the “hypersalinity first” treatment group for brackish water ecotype individuals. BW (B) is the “hyposalinity first” treatment group for brackish water ecotype individuals. FW is the treatment group for freshwater ecotype individuals.

The snails of the FW ecotype were exposed to hypersaline conditions—apart from their natural habitat conditions (0.5 PSU)—namely to 6, 12, and 18 PSU. The BW individuals were measured at the salinity of their habitat (12 PSU) and sequentially exposed to hyper—as well as hyposaline conditions. The animals were either put under hypersaline conditions first, namely to 16, 22, and 28 PSU, or to hyposaline conditions first, namely to 8, 4, and 0.5 PSU (Figure 1). The extreme salinities were chosen to represent the upper and lower limits of survival for the populations of *T. fluviatilis* used in this study. The return to 12 PSU (native salinity) without prior habituation was included to evaluate whether respiration rates would recover to baseline after exposure to extreme salinities, thereby providing insights into potential carry‐over or exhaustion effects. Two animals from each of the two ecotypes died during the experiments while being exposed to extreme salinities. Their data have not been considered in the final calculations. In total, 24 FW individuals (S1: 8 individuals, S2: 6 individuals, S3: 10 individuals) and 14 BW individuals were tested across the respective salinity treatments. The number of measurement cycles varied depending on the duration of the measurement phase and the duration of the experiment with the lowest number of repeated measurement cycles being 15 and the highest number being 59.

### 2.4 Data Analysis

Respiration rates were calculated using the software supplied by the Q‐Box AQUA Aquatic Respirometry Package (Equation 1). The data was then exported and screened for technical outliers, which were removed from the data set. Technical outliers were defined by erratic jumps in the oxygen concentration recorded; this was most likely caused by static electricity buildup over time. Any negative respiration rates were set to zero. This was done under the assumption that the snails did not respire at that time point (operculum closed) and the negative respiration (i.e., oxygen generation) was potentially caused by microalgae. This was corroborated by a measurement of the oxygen depletion in the experimental chamber containing a recently deceased snail. Means were calculated from the repeated measurement cycles per individual, and displayed as scatterplots and as a dot cloud plot with a linear mixed‐effect regression model (LMER) fitted. The means used in the LMER were square‐root‐transformed to achieve a normal distribution of the data. The dot cloud plot was generated using R version 4.3.0 utilizing packages lme4, lmerTest, tidyverse, and ggplot2 (Bates et al. 2015; Kuznetsova et al. 2017; Wickham 2016; Wickham et al. 2019). Scatterplots were generated utilizing the R package ggplot2. The initial linear mixed‐effects regression models included salinity and weight as fixed effects, with sex as a random effect. For the BW ecotype, treatment group, and its interaction with salinity were incorporated as additional fixed effects (Table 1). Following the initial model fitting, the step function (R Core Team 2023; R package stats version 4.3.0) was applied to identify the best‐fitting model based on the Akaike information criterion (AIC).

**Table 1:** Initial and final linear mixed‐effect regression models (lmer) for freshwater and brackish water ecotypes.

Freshwater ecotype				
Initial model: lmer (respiration rate ~ salinity + body weight + [1| sex])				
Final model: lm (respiration rate ~ salinity + body weight)				
Fixed effects (FW)	Estimate	Std. error	Pr (> |t|)	
Intercept	5.59773	0.39254	< 2e−16***	
Salinity	−0.05929	0.02055	0.004885**	
Weight	−10.21240	2.92337	0.000742***	
Brackish water ecotype				
Initial model: lmer (respiration rate ~ salinity * treatment group + body weight + [1| sex])				
Final model: lm (respiration rate ~ treatment group)				
Fixed effects (BW)	Estimate	Std. error	Pr (> |t|)	
Intercept	6.9523	0.6145	< 2e−16***	
Treatment group	−1.4418	0.4112	0.00067***	

*Note:* Intercept shows the estimated respiration rate when salinity and weight are both zero. The estimate represents the effect size of each predictor in the model. The *p*‐value (Pr (>|t|)) indicates whether the predictor variable has a significant effect on the response variable. Significance values: *p* < 0.001 “***”; 0.01 “**”; 0.05 “*”.

Equation 1: Equation used to calculate respiration rate (mg/kg/h).

(1) $${\mathrm{VO}}_{2}=\text{DOslope}\;*(\mathrm{Vr}-\mathrm{Va})*60/m-\text{Background}\;{\mathrm{VO}}_{2}$$

VO_2_ oxygen consumption (mg kg^−1^ h^−1^).
DO slope rate of decrease of dissolved oxygen (mg L^−1^ min^−1^).
Vr respirometer volume (mL).
Va volume of experimental animal (mL).
m animal weight (g).
Background VO_2_ measured with an empty chamber.

## 3 Results

**Figure 2:**
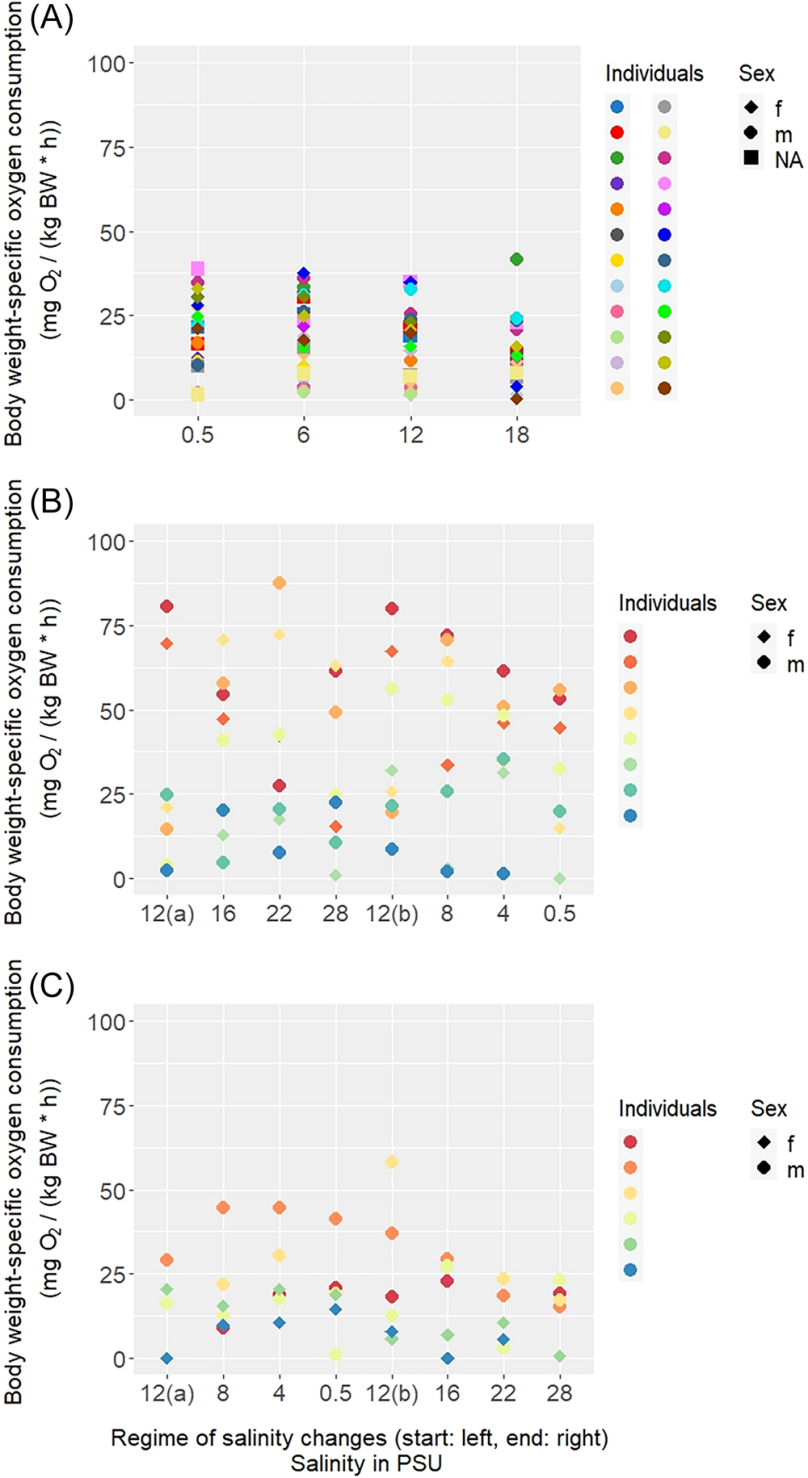
Body weight‐specific respiration rates in snails exposed to media with different salinities. Each dot represents the mean respiration rate of an individual snail. The oxygen consumption rates of the animals exposed to media with the salinities of their original habitats at the start of the experiment are shown on the left of each panel: 0.5 PSU for the FW animals or 12 (a) PSU for the BW animals, respectively. The salinity 12(b) shows the respiration rates of snails exposed to a medium with the salinity of their original habitat after going through either “hyposalinity first” or “hypersalinity first” treatments, respectively. (A) Snails of the freshwater ecotype, *n* = 24, except at 12 PSU: *n* = 23, and 18 PSU: *n* = 22. (B) Snails of the brackish water ecotype in the “hypersalinity first” treatment, *n* = 8, except at 0.5 PSU: *n* = 7. (C) Snails of the brackish water ecotype in the “hyposalinity first” treatment, *n* = 6, except at 12(a) PSU: *n* = 4, and 28 PSU: *n* = 5.

**Figure 3:**
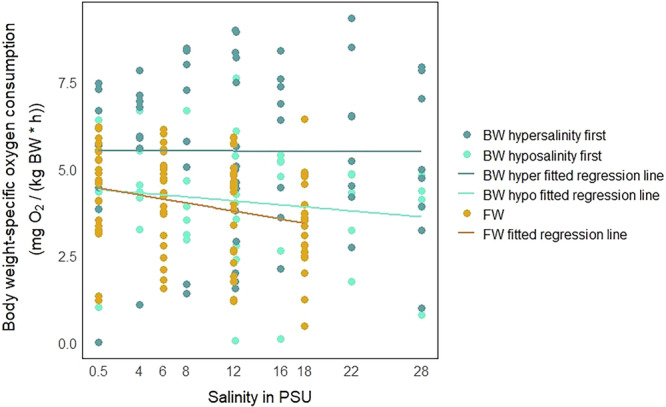
Mean respiration rates in freshwater and brackish water individuals of *Theodoxus fluviatilis* exposed to different salinities. Each dot represents the mean respiration rate of an individual snail. Data were square root transformed (sqrt). Color code: Ocher for freshwater, green for brackish water individuals. Dark green is for animals in the hypersalinity treatment group, and light green is for animals in the hyposalinity treatment group. The bright green line represents the linear mixed‐effect regression model (lmer) for the hyposalinity first treatment of the BW ecotype. The dark green line represents the lmer for the hypersalinity first treatment. The ocher line represents the lmer for the FW ecotype.

The differences in respiration rates were displayed in scatterplots and analyzed using a LMER. The data was displayed as body weight‐specific oxygen‐consumption rate per individual at each of the tested salinities (Figure 2). The animals of the FW ecotype were only tested under hypersaline conditions (Figure 2A). The respiration rate decreased with increasing salinity (Figures 2A and 3). For the FW ecotype the linear mixed‐effects model shows that salinity has a significant effect (*p* = 0.004885) on the respiration rate of the individuals. Additionally, according to the model, the weight of the individuals also has a significant effect on the respiration rate—the bigger the individual, the lower the respiration rate (Table 1). The animals of the BW ecotype were split into two treatment groups. The “hypersalinity first” treatment group (Figure 2B) showed generally higher respiration rates in comparison to the “hyposalinity first” treatment group (Figure 2C) and also in comparison to the FW animals (Figure 2A). According to the linear mixed‐effects model, the respiration rates of the brackish‐water animals were significantly affected by the treatment group, not, however, by the change in salinity (Table 1). This indicates that the directionality of salinity treatments has an impact on the respiration rate, meaning the sequence of exposure to varying salinities influenced oxygen consumption more strongly than the absolute salinity level. The possibility of the treatment group masking a salinity effect was tested for by removing the factor “treatment group” from the model. However, this made no difference in terms of statistics, and we concluded that masking of the salinity effect did not occur. Respiration rates did not significantly differ between males and females. The baseline‐respiration rates at the original habitat salinities also didn't show significant differences between the FW and BW ecotypes (Figure 2). In contrast to the BW individuals, animals of the FW ecotype showed a clear decrease in respiration rates with increasing salinities as shown by the different slopes of the regression lines of respiration rates over all salinities (Figure 3).

## 4 Discussion

The ability to adequately respond to rapid changes in salinity in the surrounding medium is crucial for slow‐moving organisms such as gastropods living in shallow coastal waters. Heavy rains may lead to decreases in salinity, while intense sunshine may result in rapid increases in local medium osmolality. Salinization of shallow FW ponds in the course of climate change also poses challenges to FW snails (Velasco et al. 2019; Venâncio et al. 2019). Additionally, water run‐offs during winter can lead to a salt influx into lakes and streams, caused by road salt (Szklarek et al. 2022). Changes in external salinity exert direct effects on the metabolism of affected molluscs and also result in changes in behavior (Mann et al. 1991; Strömberg and Spicer 2003).

In this study, we examined the oxygen consumption (respiration rate) as a proxy for overall metabolic rate in individuals of two different ecotypes of the neritid snail *T. fluviatilis*. Body weight‐specific baseline respiration rates did not significantly differ between individuals of the FW and the BW ecotypes (Figure 2). This finding corresponds to results of a previous study on the oxygen consumption of *T. fluviatilis* collected from BW in Denmark (Lumbye 1958). This allows the conclusion that the baseline metabolic rate of specimens of the two *T. fluviatilis* ecotypes, although living in very different salinity conditions (FW: 0.5 PSU, BW: 12 PSU), is quite similar.

Increasing the salinity of the surrounding medium prompted the FW snails to decrease their respiration rates by about 0.6 mg O_2_/(kg body weight × h) per ‰ salinity increase (Figure 3). This corresponds to observations in the FW gastropod *Chilina dombeiana*, which was reported to reduce respiration rate when exposed to BW (12 PSU) (Barrios‐Figueroa and Urbina 2023). Unfortunately, in our experiments, it was not possible to monitor the behavior of the snails in the respirometry chamber, but decreasing physical activity or even closing of the operculum during exposure of animals to high salinities may have contributed to this effect.

A decrease in respiration rate with increasing body weight has been shown for a variety of organisms (Von Bertalanffy and Pirozynski 1953; Ikeda 1970; Marsden et al. 2012). The BW ecotype individuals' sizes ranged from 0.07 to 0.019 g, while the FW ecotype individuals' sizes were much more variable with the smallest being 0.023 g and the biggest 0.191 g. This might be the causal explanation why the weight didn't have an impact on the oxygen consumption of the BW ecotype according to the linear mixed‐effects regression model.

While weight and salinity changes significantly affected the respiration rates of the FW ecotype, the respiration rates of the BW ecotype individuals were not so clearly correlated with changes in medium salinity (Figure 3; about 0.01 mg O_2_/[kg body weight x h] per ‰ salinity increase), but were, in tendency, dependent on the directionality of the treatment (Figure 2B,C, Table 1). However, the respiration rates of the BW individuals generally showed a higher interindividual variability over all treatments compared with those of the FW individuals (Figure 2). This might be due to a higher genetic variability in individuals of the BW ecotype, caused by adaptation to the variable Baltic Sea shore environment. While differences in physiological conditions or osmolyte reserves can't be definitively excluded as reasons for the higher interindividual variability, it seems unlikely to be the cause since all individuals (BW and FW) were treated the same before and during the experiments. Additionally, the respiration rates of the “hypersalinity first” treatment group showed greater inter‐individual variation than the individuals in the “hyposalinity first” treatment group (Figure 2B,C). However, the intra‐individual variability of the respiration rates between “hyposalinity first” and “hypersalinity first” treatment groups were similar (Figure 2B,C).

Exposing the BW individuals to a broad range of salinities does not significantly affect the respiration rates in either the “hyposalinity first” or the “hypersalinity first” treatment (Figure 3). However, it seems (Table 1) that the sequence of treatments (“hypersalinity first” or “hyposalinity first”) may have an impact on the general level of oxygen consumption in the BW animals. When comparing oxygen consumption data shown in Figure 2B,C, it appears as if the respiration rates for the “hypersalinity first” treatment group (Figure 2B) are tendentially higher—even in the low salinities‐ compared to the group exposed to the “hyposalinity first” treatment (Figure 2C). This trend is confirmed by the mixed‐effects regression model, which shows that the respiration rate of the BW ecotype is significantly influenced by the direction of treatment (Figure 3, Table 1).

Considering the usual challenges of the animals of the BW ecotype in the wild, it seems not surprising that hyperosmotic conditions seem to be more stressful for the animals in comparison with hyposaline conditions. As previously reported (Wiesenthal et al. 2019), the BW animals accumulate high amounts of newly synthesized organic osmolytes (amino acids and urea) in their body fluids to avoid tissue shrinking under hypersaline conditions. All of these actions require energy (Pierce and Amende 1981), which may explain the generally higher respiration rates in animals receiving the “hypersalinity first” treatment. It may be speculated that these osmolytes are at least in part actively released to the ambient medium when the animals are subsequently exposed to hyposaline conditions to avoid tissue swelling by osmotic water influx. Such a mechanism has been previously described for the bivalve *Modiolus* under similar conditions (Pierce 1971). This would be associated with a substantial loss of energy‐rich molecules that cannot easily be replaced from internally stored organic molecules. The resulting “material exhaustion” may explain why the respiration rate of individuals having gone through a period of exposure to hyposaline media does not recover when the animals are exposed to their original medium (Figure 2C, data point 12(b) PSU) or to even higher medium salinities (Figure 2C, data points 16, 22, and 28 PSU).

## 5 Conclusion

This study examined the respiration rates of FW and BW ecotypes of *T. fluviatilis* in response to changing medium salinities. While baseline metabolic rates were similar, FW snails reduced respiration when exposed to hypertonic media. BW snails exhibited greater interindividual variability in oxygen consumption at basal altered salinity conditions and a wider salinity‐tolerance range than the FW animals. Moreover, oxygen consumption was affected by the experimental regime, and was generally higher in animals initially exposed to hypersaline media compared to those initially exposed to hyposaline media. The results indicate that the provision of organic osmolytes in the tissues of animals exposed to hypertonic media is costly in terms of energy and materials. When animals are exposed to hypotonic media, they may rapidly release such osmolytes to the environment, which results in a relative exhaustion of their capabilities of tissue‐ and cell‐volume regulation. This study further highlights the physiological differences between the FW and BW ecotypes of *T. fluviatilis* in northern Germany and creates an incentive to investigate the possible genomic basis of these.

## Conflicts of Interest

The authors declare no conflicts of interest.

## Data Availability

All relevant data and supplementary information can be found within the article. Raw data files are available at www.figshare.com. Raw respiration data for BW ecotype: https://doi.org/10.6084/m9.figshare.25575249, raw respiration data for FW ecotype: https://doi.org/10.6084/m9.figshare.25576854.
